# Hormonal stories: a new materialist exploration of hormonal emplotment in four case studies

**DOI:** 10.1057/s41292-023-00317-8

**Published:** 2024-01-28

**Authors:** Sonja Erikainen, Andrea Ford, Roslyn Malcolm, Lisa Raeder

**Affiliations:** 1 Department of Sociology, School of Social Science, University of Aberdeen, Edward Wright Building, Dunbar Street, Aberdeen, AB24 3QY Scotland; 2 Centre for Biomedicine, Self and Society, Usher Institute, The University of Edinburgh, 23 Buccleuch Place, Edinburgh, EH8 9AG Scotland; 3 Department of Anthropology, Durham University, Dawson Building, South Road, Durham, DH1 3LE UK

**Keywords:** Hormones, Emplotment, Narrative, Stories, New materialism

## Abstract

Hormones are complex biosocial objects that provoke myriad cultural narratives through their association with social activities and identities, and these narratives have the power to shape people’s lived realities and bodies. While hormones were historically conceptualised as ‘master molecules’ capable of controlling various life processes, their explanatory potential has now been overshadowed by technoscientific developments like omics- and gene-based biotechnologies that have reframed how human bodies and behaviours are understood. Considering these shifts, this paper asks what roles hormones perform and what stories they are arousing today. Through a patchwork of four hormone stories about contraception, gender hacking, birth, and autism-specific horse therapy, we show how hormones remain vital protagonists in the constitution of bodies, affects, environments, places, politics, and selves in the contemporary period. Building on new materialist approaches, we adopt and extend the notion of ‘emplotment’ to encapsulate how hormones act as key characters in our plots. They are working to complicate dominant understandings of what bodies are and can be in new ways as they mediate different plots of bodily experience, in ways showing the ongoing powerful salience of hormones and their ascendancy in the present.

Hormones are central characters that perform multiple roles in different scientific, social, personal, and political stories. In biomedical narratives, hormones are generally a class of signalling molecules that travel through the circulatory systems of multicellular organisms, and provoke, regulate, or appease a broad variety of physiological and behavioural responses (Oudshoorn 1994; Fausto-Sterling 2000; Roberts 2007). They play key roles in life processes like reproduction, respiration, digestion, metabolism, sensory perception, sleep, and mood, as well as internal homeostasis itself. Indeed, historically, hormones were initially conceptualised as ‘master molecules’ that could control these myriad life processes, and thus they represented the possibility of gaining control over and improving life via hormonally manipulating human bodies and behaviours (Rasmussen 2002; Nordlund 2007).

As these early conceptualisations suggest, hormones are also socio-cultural entities that provoke a variety of cultural narratives through their association with social activities and identities. For example, popular articulations of love and social bonding commonly position oxytocin as a central character (Malcolm 2021). Similarly, serotonin has been constituted as biological correlate of happiness, and cortisol as the chemical manifestation of stress (Roberts & McWade 2021). Hormones’ place in socio-cultural phenomena also makes them actors in stories that shape ethical and regulatory oversight. Testosterone, for example, has not only been seen as the essence of masculinity and as a key actor in sports success, but testosterone levels have also been used to regulate athletes’ right to compete in international sport competitions, while doping with testosterone is often framed via a story of unfairness and moral wrongdoing that warrants a regulatory ban (Erikainen 2019). Hormones have become important actors in narratives about wider environmental systems as well, from endocrine disrupting chemicals in manufactured goods to pharmaceuticals in food supplies (Langston 2010; Ah-King and Hayward 2013; Pollock 2016; MacKendrick and Cairns 2019).

Through the many stories that they provoke, hormones mediate the nexus of biomedical, socio-cultural, environmental, personal, political, and regulatory spheres of life, making them complex biosocial objects. Today, in the social sciences and humanities, new theoretical interventions and especially new materialist frameworks have worked to reconfigure hormones as active and agentic entities that have the ability to ‘provoke’ social, cultural, and political as well as biological phenomena, in intertwined ways. Concurrently, while social scientific and humanities scholarship has long theorised narrative as a central way that social actors make sense of the world, the storied nature of meaning making has also been applied directly into the realm of action through concepts such as ‘emplotment’ (Mattingly 1994). This shows how the construction of plots is a key way actors carry out and make meaning from their actions, and particularly in relation to narratives about health and the body. With new materialist insights, it is also possible to examine what happens to such narratives when phenomena like hormones are configured as active agents in the emplotment of people’s lived realities.

In the last few decades, the early ‘master molecule’ hormone narratives have come to be overshadowed by new technoscientific developments such as molecular genetics and other forms of gene-based biotechnology. Yet, hormones continue to have a profound influence on people’s lived experience and inhabited worlds. They still carry the power to influence large-scale policy decisions as well as intimate feelings about one’s personal experiences as they flow across multiple spheres of life. They are a continual site of ‘re-making’ whereby new stories are attached to and reworked by their movements through myriad scales of scientific, popular, regulatory, and personal developments. In this socio-cultural and technoscientific context where hormonal models of the body sit alongside (and sometimes intersect with) new and emerging technoscientific frameworks like genomics and other omics—biomedical technologies that have reframed how human bodies and behaviours are understood—it is worth asking the question, what kinds of roles do hormones perform in our contemporary narratives of life? As endocrinological knowledges themselves are consumed, (re)constructed, and contested, what kinds of protagonists are hormones today in stories that are told about bodies, selves, and the environments they inhabit? To the extent that the ‘master molecule’ visions of hormones were surpassed for a period of time by new biomedical explanatory models, what kinds of stories do hormones ‘arouse’ or ‘put into action’ in the present context?

In this paper, we consider these questions via four different research projects in conversation with new materialism, drawing on qualitative data in the form of participant observation, semi-structured interviews, media articles, commercials, websites, and other socio-cultural artefacts that examine the biosociality of hormones. These research projects were conducted within the ethical approval frameworks of our respective universities. We build on new materialist conceptualisations of hormones as active agents that perform key roles in the narrative emplotment of lived realities by tracing different albeit overlapping ways in which hormones act as protagonists in the contemporary world. Firstly, we show how hormones act as agents of control by examining the gendered ways in which they influence control narratives around hormonal contraceptives, while also arousing affective responses. Secondly, we examine how hormones reconfigure the gendered body by looking into narratives around ‘gender hacking’ where hormones are key protagonists in future-oriented narratives about re-making gendered bodies and social relations. Thirdly, we consider how hormones enact both ‘the natural’ and ‘the medicalised’ as plotlines in birth, through examining how birth hormones are understood as key protagonists inciting cascades of effects in childbearing bodies. Finally, we inquire into the ways in which hormones sit at the nexus of body-person-worlds as environmentally situated relational agents, by considering how hormones link the body, the self, and the environments they inhabit in the context of autism-specific horse therapy.

By bringing these research threads together, we argue that hormones remain vital protagonists in the constitution of bodies, affects, environments, places, politics, and selves today. They mediate between dominant and alternative plots of bodily experience, figuring as key agents in narrative emplotments of lived realities in ways that challenge dominant understandings of bodies. Alterative emplotments of what hormones are and what they can do work to challenge dominant biomedical models of hormones and how people ‘fit’ within these models, at the same time as people are using these alterative emplotments to ‘hack’ their bodies by tinkering with hormones. In so doing, they are problematising dominant biomedical models of sexed, gendered, ‘natural’ and ‘biological’ bodies as well as how these bodies and embodied processes become situated in their various environments and contexts.

## Configuring hormones through new materialism

In the first half of the twentieth century, hormones were figured as life’s ‘master molecules’, representing the prospect of power over the processes of life as well as vast commercial gains from bringing hormone therapies to market (Nordlund 2007; Rasmussen 2002). Hormones were conceived of as master controllers of life processes, such as respiration in the case of hormones in the adrenal cortex, for example (Rasmussen 2002). The expectation was that it would be possible to not only understand human life through analysing hormones, but that endocrinologists would also eventually be able to improve or enhance life through hormonal interventions, including by changing human bodies and behaviours (Nordlund 2007).

This early ‘master controller’ narrative has especially been analysed in the context of sex endocrinology, where the framing of oestrogen and testosterone as the ‘essence’ of femininity and masculinity, respectively, sparked a hormonally constructed conceptualisation of the body (Oudshoorn 1994; Roberts 2007). This became not only a central model of thinking about embodied sex and gender difference as hormonally induced but also enabled the commercialised development of oestrogen and testosterone into the most widely used drugs ever produced (Oudshoorn 1994). Indeed, as Roberts (2007, p. 26) has observed, the so-called ‘sex hormones’ have been and continue to be understood as central to the (re)production of sexed and gendered bodies, and they are, thus, still “key actors in the production of contemporary ways of being”. Since the second half of the twentieth century, however, the ‘master molecule’ visions around hormones have in many ways been surpassed by molecular genetics and other forms of gene-based biotechnology (Rasmussen 2002; Nordlund 2007).

In recent decades, social science and humanities scholars have applied new materialist theorising to understand sex hormones in ways that aim to disrupt and move beyond conceptual divisions between the social and scientific, and biological and cultural. New materialist thinking emphasises the agency and ontological instability of materiality itself, with thinkers like Haraway (1991), Kirby (1997), Barad (2007), and Hird (2019) working to rethink the ontology of matter as active, fluid, and shifting rather than inert, fixed, and stagnant, through an engagement with objects conventionally figured as the purview of the natural sciences. In relation to hormones, new materialist frameworks enable hormones to be seen as ‘radically relational’ due to the way in which they work with other entities and flow across conventional conceptual and epistemic boundaries (Roberts 2007).

Etymologically, ‘hormones’ are those which ‘excite’, ‘arouse’, or ‘put into action’—a potent metaphor not only for their physical, behavioural, and psychological effects, but also their relation to social and cultural stories. As Roberts (2007) argued, hormones are configured as ‘messengers’ but the questions of exactly what messages they carry and to whom have answers that exceed biological frameworks. They carry not only messages that stimulate cells to create proteins, arousing physiological responses within closed bodily systems, but also, concurrently and in intertwined ways, social and cultural messages that arouse meaning, shaping identities and experiences. Hormonal action cannot be understood without the social and cultural frameworks that make their biological actions meaningful, but they also act directly within the social and cultural, making them “active agents in bio-social systems that constitute material-semiotic entities” (Roberts 2007, p. 22). They are thus a key example highlighting the need for such conjoined neologisms as biosocial and material-semiotic, which have become ubiquitous in medical humanities and social science. As Irni (2013) has argued, one should thus analyse not only how different *bodies* are affected by hormones but also how *people* are moved or ‘affected’ by them, including the affective dynamics of these hormones in forming a sense of self, and the wider social apparatuses that both enable and restrict the enactments that hormones can perform.

These newer frameworks propose an ontology of hormones that highlights their ability to ‘provoke’ phenomena beyond the biological, extending their sphere of activity and influence to the social, cultural, and political. We build on these theoretical framings, centring the narratives that hormones ‘provoke’ as the focus of our analysis. In so doing, our intention is to consider stories as, to borrow from Irni (2013, p. 54), “material-discursive ways in which … hormones … actively work in society”.

## Narratives, stories, and plots; understanding hormones through narrative modes of provocation

Narrative is deeply ingrained in the conceptual landscape of the humanities and social sciences. Scholars like Plummer (1995) have demonstrated how social subjects use stories to ‘make meaning’ or to ‘give sense’ both to themselves and the world around them, responding to narratives about others’ experience with narratives of their own (Smith and Sparkes 2011). Stories have been theorised as a powerful way of making sense of and imposing order on wider social and cultural systems and processes, including scientific knowledge production. For example, social and cultural narratives are used and appear throughout biomedicine, showing how scientists produce knowledge by telling (often gendered and racialised) stories about phenomena as varied as molecules, conception, different organs, and the immune system (see e.g. Martin 1991; Haraway 1991). These scientific stories travel unevenly into public understandings and media portrayals, which influence people’s behaviours and understandings of bodies in the world. As understandings of situated bodies change, scientific narratives overlap with, and also diverge from, popular stories and ‘alternative’ expertise, particularly in areas of health. Martin (2001), for example, explores this in her study of the immune system and metaphors of flexibility at the turn of the century, demonstrating how scientific narratives are merged with and interpreted in relation to other cultural and political narratives—in this case, neoliberal corporate organisation. Hormones, similarly, are ‘made’ and ‘re-made’ through overlapping and cross-fertilising scientific and cultural stories, which help us make sense of what hormones are and what they can do (Erikainen et al 2020).

Some scholars have carried the storied nature of meaning making directly into the realm of action. Through her use of the notion of ‘emplotment’ in the context of therapeutic encounters, Mattingly (1994) argues that narrativity, and especially the work of constructing plots out of successions of actions, is a key part of how actors carry out and make meaning out of social action. Social actors are motivated to ‘plot’ their actions into a coherent narrative structure, creating a whole out of a sequence of events, because particular actions take on their meaning by contributing to a larger, unfolding story. It is this ‘making whole’ that creates meaning: actions take on significance when recognised as a part of a wider story that is being constructed. Processes of emplotment are multiply authored—there is no single storyteller but a plurality of actors and actions. In Mattingly’s rendering of emplotment, medical professionals and patients were central actors. In this article, we build on new materialist thinking around the entwinement of the material and cultural, and examine what happens to the notion of narrative when phenomena like hormones are configured as central actors in the emplotment of lived realities. Existing analyses of hormonal narratives serve as a basis for this work, an important example of which is Jordan-Young and Karkazis’ (2019) analysis of ‘T talk’, which can be understood as social, political, and biomedical storytelling about testosterone. ‘T talk’ weaves folklore into science and science into cultural beliefs, shaping what testosterone becomes, “both as a material substance and as a multivalent cultural symbol” (Jordan-Young and Karkazis 2019, p. 10). Further, some new materialist thinkers have rethought the construction of narratives through extended notions of textuality where materiality is figured as a kind of ‘writing’, and the body (whether human or otherwise) as a scene of writing (e.g. Kirby 1997). Within these kinds of conceptualisations, bodies as well as phenomena like hormones can not only be written but they can also ‘write back’ (Kirby 1997), which in turn requires a way of reading materiality that allows it to alter our narratives, making them unstable and shifting.

Examples of this line of thinking are Haraway’s figurations of resistant ontology, including the cyborg (1991) and Oncomouse (1997) but also figurations such as ‘the human genome’, which turn “body into story, and vice versa, producing both what can count as real and the witness to that reality” (1997, p. 179). Haraway’s figures function as alternative and potent ‘emergent realities’ that enable newly possible bodies, and give rise to a politics of ‘writing otherwise’ that retells dominant narratives about reality through these material-semiotic figures (Haraway 1991). Some have taken this onwards through thinking hormones as “forms of social, material, and biological writing” by working with an “expanded notion of what constitutes reading and writing” (Dickinson 2019). For example, in his work on “metabolic poetics”, Dickinson (2019, 2018) examines endocrine-disrupting chemicals to inquire into how contemporary writers might respond to the capacity of these chemicals to concurrently provoke social as well as biological formations. In his expanded notion of writing, narrative forms such as poetry can function as productive hormone disruptors within the context of wider cultural narratives about hormone disruption. We can concurrently ‘read’ the chemicals in bodily fluids that are materially interfering with our human endocrine systems and rewriting our bodies’ biochemical messages, meaning that in ‘biosemiotic’ terms, endocrine-disrupting chemicals can be understood as a form of writing, including as a form of rewriting our bodies.

In what follows, we build on these conceptualisations to examine the dynamism of hormones’ provocations by exploring what shared threads emerge when we consider hormones as key actors in multiple narratives, read together. We consider how hormones act in and across different sites today, both materially and semiotically, simultaneously maintaining old ‘master molecule’ narratives while problematising them. In bringing together insights across four different stories featuring hormonal protagonists, we are interested in the roles that hormones play in the constitution, and engagements, of biological, social, political, spatial, affective, and embodied realities in the contemporary world. In our examples, hormones remain protagonists through (1) acting as agents of control, (2) reconfiguring the gendered body, (3) re-articulating the enduring natural-versus-cultural binary, and (4) making the body a place of congenial social relations through external therapies that hail internal entities. We demonstrate how hormones’ provocations flow in, through, and across multiple ‘realities’ in ways that both solidify and obscure their analytic boundaries.

## Hormones as agents of control: hormonal contraceptives

Raeder’s work on the biosociality of hormones, drawing on qualitative interviews conducted in 2020 with Swedish women who had participated in a contraceptive consultation at a sexual health clinic in Stockholm, shows how hormones both provoke and disrupt narratives of control relating to hormonal contraception, in ways that arise and mobilise affect in relation to gendered practices of hormonal self-regulation. Hormonal contraceptives are, concurrently, biotechnologies for medical regulation and production of gendered bodies, and cultural artefacts producing social and cultural relations, practices, and subjectivities (Roberts 2007). In biomedical narratives, hormonal contraceptives are commonly figured as tools enabling women to take control of their bodies and fertility, but also as tools for managing health more generally, producing femininity, and achieving sexual emancipation and gender equality (Bertotti et al. 2021; Preciado 2018; Reed and Saukko 2010; Mamo and Fosket 2009; Granzow 2007). In the contraceptive stories of Swedish women interviewed by Raeder, hormones are central protagonists and key actors in the narratives about control and behaviour regulation that they emplot, which show how hormones both engender and unsettle affective experiences of control over bodily processes and of selves.

At the time of their interviews, Tove and Emelie were both using a hormonal contraceptive method—Tove the pill, and Emelie the vaginal ring. They state that a contraceptive priority for them is that their contraceptive method “affects them as little as possible”, and that they are able to “feel like themselves”, suggesting that hormonal contraceptives provoke not only bodily effects but also affective changes in one’s sense of self that are experienced as undesirable. They both express uncertainty about the extent to which their chosen method affects them beyond regulating fertility and menstruation; an uncertainty they respond to by pausing use to track potential changes in their health and sense of self. Tove explains that taking a break from hormonal contraceptives provides her with experiential feedback, enabling her to feel more aware of how she’s doing on her contraceptive method. Periods of non-use are here described as a tool enabling her to monitor her health and self, suggesting that it is the practice of taking a break to “check in” on her body and health that results in affective responses of feeling calm and in control of herself as well as her hormonal regimen rather than the contraceptive method itself.

Indeed, being able to stop and resume use is narrated by the participants as an important aspect of exercising control of oneself and one’s body and health—control which is enacted via hormonal contraceptives both through their use and discontinuing it. The notion of discontinuation as a form of control diverges from mainstream biomedical narratives of hormonal contraceptives, where control is understood to be exercised through continuous use; a framing primarily based on the metric of efficacy (Bertotti et al. 2021) as well as on biomedical framings of female bodies, fertility, and the management of reproduction. This is centrally connected to the ways in which hormonal contraceptives affectively shape sense of self due to how they act on the body to alter its processes. Emelie stresses the urge to keep track of her sense of self, emphasising the difficulty in understanding the extent to which her body and health is affected by her contraceptive method:

You know, it feels like… There is something in me that finds all this a bit strange. Because I know that it affects me. And how much do I want that? What is it that affects me and how much?

Tove reflects on what she considers most important in her contraceptive method besides regulation of fertility:

That it affects me as little as possible, which is… I mean, in a way that you don’t have to think about it so much. And you might think that having a hormonal IUD would be great then, as I didn’t menstruate at all while I had the IUD. But instead that then became something that occupied my mind, questions like ‘am I pregnant?(…) And on the Pill, thoughts like ‘What do I do now when I forgot to take the Pill one day and I immediately started bleeding, but I still have pills left to take?’. It means that I have to work out ways of managing my Pills. (…) I just want to be able to carry on as usual without my contraception affecting my life.

In both Emilie’s and Tove’s narrations, the ways in which hormonal contraceptives act on their bodies to alter its processes are experiences as a site of uncertainty, and the contraceptives themselves gain an aura of a potentially unknown entity acting upon the body and self that one must then actively try to understand and control. Unlike in biomedical narratives where hormonal contraceptives provide women with control over fertility and menstruation, hormonal contraceptives’ provocations on the body here emerge as something that gives rise to *loss* of control over one’s bodily responses to the contraceptive method. The management of uncertainty produced in experiences of hormonal contraceptives is depicted as work, further troubling the biomedical narrative of hormonal contraceptives as biotechnological tools for stabilising fickle bodily processes and generating predictability, helping users live life with less worry about their reproductive health (Bertotti et al. 2021; Mamo and Fosket 2009).

Felicia says that hormonal contraceptives have been part of her everyday life since being a teenager. Due to severe period pains and PMS, she has used various hormonal contraceptive methods, only pausing use when she and her partner decided to have children. When resuming use of the vaginal ring, she discovered that her contraceptive method was not working “as it should”. Here, ‘as it should’ is for Felicia not referring simply to avoiding pregnancy or allowing her to skip periods, but rather, it enables her to manage PMS symptoms and to “feel well”. Instead, she describes how her contraceptives also provoked side effects such as weight gain, rashes, and depression. At the time of the interview Felicia had stopped using the vaginal ring and expressed a sense of relief to be “hormone free”:

I think I’ve gotten to the point in my life where I felt like I don’t have the energy to experiment so much with my body anymore. You’ve been pregnant a lot, and hormones spouting all over (…) after so many years of either being on contraceptives or being pregnant, it’s just nice to get to land in ‘This is my body, this is how it works without things you put in it that affects you.’

Like in Emilie’s and Tove’s narratives, in Felicia’s account, hormonal contraceptives emerge as potentially unknown or foreign substances that affect the body, altering how it works in ways experienced as unpredictable, and resulting in the need to manage this unpredictability. Further, the fact that these contraceptives provoke not only effects that are desired, including avoiding pregnancy and managing PMS, but also side effects that are not desired speaks to the ways in which hormones have their own agency. That these hormones were not working as Felicia felt they should suggest a tension between the desire to predict and control hormones’ actions, and the ability of hormones to carrying out their own actions and provoke changes in our bodies that we may not want them to. For Felicia, the implications of this were that gaining a sense of control of her body and health meant, at this point in time, not using hormonal contraceptives, which also challenges the biomedical narrative of hormonal contraceptives as tools for enabling control, producing an alternative conceptualisations of what ‘being in control’ means. Participants’ accounts of “taking control” by not using rather than using hormonal contraceptives, or by pausing hormonal contraceptive use for periods of time, highlight a conceptualisation of control that diverges from the biomedical narrative of hormonal contraceptive methods as enablers of control. Instead, control emerges here as relational with reference to the complex affective provocations that hormones enact to shape the body and sense of self (Irni 2013).

In these women’s narratives, hormonal contraceptives thus emerge as central protagonists that act on the body and provoke not only physical, but also affective effects that highlight how meanings and enactments of hormonal contraceptives as agents of control are not ‘fixed’, but fluid and relational. Centrally, while they figure in stories about ‘control’, the notion of ‘control’ is ambivalent and shaped by the uncertainty that is generated by hormones’ ability to act on the body not only in desirable but also undesirable ways. This ambivalence maps poorly onto biomedical narratives about hormonal contraceptives as tools for women to gain ‘control’ over their reproductive bodies, because the biomedical narratives are undermined by hormones agency and thus contested by the women themselves who experience hormones acting on their bodies in ways that results in a sense of loss of control rather than gaining it. While these women drew from biomedical control narratives to make sense of their own experiences, they articulated explicitly alternative notions of ‘control’ where it was framed in terms of liberation from both hormonal contraceptives and the biomedical narratives that position these contraceptives as master controllers of women’s bodies.

## Hormones as reconfigurers of the gendered body: hormonal gender hacking and self-medication

Erikainen’s research on the narratives of so-called ‘gender hackers’, who are an emerging movement of individuals appropriating biotechnologies such as synthetic androgens and oestrogens for their own purposes, exemplifies how hormones are mobilised in and act to facilitate future-oriented narratives about the re-making of gendered bodies. Preciado (2018) has used the ‘gender hackers’ term to denote individuals and groups who use the so-called sex hormones in ways that subvert the ‘legitimate’ or ‘authorised’ uses of these hormones as proscribed by healthcare authorities. Gender hackers, he argued, are people who fall outside the naturalised binary gender system, including people who are transgender or non-binary, who “don’t identify with the term *gender dysphorics*”, and are, instead, “*copyleft* users who considers hormones free and open biocodes, whose use shouldn’t be regulated by the state or commandeered by pharmaceutical companies” (Preciado 2018, p. 55, original emphasis).

The notion of ‘gender hacking’ has gained purchase within activist communities falling under the emerging biohacking movement, which is a community-led movement of people who often do not have scientific credentials but are nonetheless undertaking biomedical experiments, including self-experimentation. They generally have political aims that challenge conventional notions of scientific and medical expertise and operate with a democratising ethos to make the present and future of biomedicine open to those it impacts, irrespective of professional status or education (see e.g. Davies 2017; Delgado 2013; Delfanti 2013). Gender hacking can be seen as a sub-movement of the wider biohacking movement that foregrounds trans and non-binary gender emancipatory politics, especially around hormones, where hormones are used and act to enable the emplotment of alternative futures of gendered bodies.

Central element of the politics of gender hacking are promissory narratives that enable the envisioning of more gender emancipatory futures for gender diverse people. New technoscientific developments generally tend to be characterised by future-oriented narratives whereby possible futures are politically manufactured and attached to the new developments as their ‘promise’ (Brown et al. 2000). These narratives tend to construct desired or desirable futures that new technologies are envisioned as capable of bringing forth. They also tend to be performative, shaping the direction of development and use of emerging technologies in ways that are geared towards the realisation of the promissory narratives that have been manufactured around them (Brown et al. 2000). Gender hacking narratives are counter-hegemonic and promissory in ways that offer radical accounts of what hormones can do and how they can or should be used, enabling hormones to carry the potential to incite reconfigurations of gender beyond the binary female versus male modalities. Gender hackers engage in material practices of hacking into social norms and (binary) gender structures through tinkering with hormones, and advocate for the vandalism or sabotage thereby inflicted upon the hegemonic binary gender system. Hormones, in turn, act as central agents of the vandalism and sabotage that is being carried out.

The Open Source Estrogen project, spearheaded by the artist Mary Maggic, combines biohacking with speculative design to examine the feasibility and implications of non-professionals, including trans, non-binary, and other gender diverse people, synthesising oestrogen themselves. The aims include developing “a set of tools, protocols, and wetware for low-cost, accessible participatory estrogen hacking” that can function as “social resistance, as consciousness raising, as DIY therapy”, and “as gender hacking”, including resistance to the way in which “institutions and scientific fields produce fictions about how bodies should be gendered … perpetuating a standard of normalcy that is exclusionary” (Hackteria 2019). Similarly, the Laboria Cubonics (2015) collective has issued a manifesto, where hormones carry the potential for ‘hacking’ the medicalised binary gender system, asking whether this potential could be harnessed to build a different kind of gender emancipatory future:

Hormones hack into gender systems possessing political scope extending beyond the aesthetic calibration of individual bodies. … We ask whether the idiom of ‘gender hacking’ is extendible into a long-range strategy, a strategy for wetware akin to what hacker culture has already done to software—constructing an entire universe of free and open source platforms … Can we stitch together the embryonic promises held before us by pharmaceutical 3D printing (‘reactionware’), grassroots telemedical abortion clinics, gender hacktivist and DIY-HRT forums … to assemble a platform for free and open source medicine?

These emplotments of the power of hormones can be seen as a form of future world-making advanced through a merger of biomedical, artistic, and textual experimentation that constructs promissory narratives about hormones’ potential to reconfigure the gendered world, and about the means to get there. Hormones—especially oestrogen and testosterone—act as ‘messengers of sex’ (Roberts 2007) but they do so specifically through their ability to carry messages both within and about sexed bodies that transgress the binary gendered organisation of bodies and social relations. If used in ‘unauthorised’ ways by gender diverse people themselves to reconfigure gendered bodies, such as through the administration of self-synthesised oestrogen by trans feminine people, hormones could arouse both physiological changes within individuals’ bodies and socio-cultural shifts in the conceptualisation of gender as a binary reality grounded upon the physiology of the body. The notion of hormones as ‘hacking’ into gender systems, through an analogy with computer hacking, is about the ability of hormones to gain an unauthorised access into an established system—the gendered body and the gendered system of social organisation—with the intention to undermine its security and stability. In this sense, hormones are recruited to act as central saboteurs of both the binary gender system in general and the medical establishments that control it through controlling ‘authorised’ access to hormones in particular. They thus function as agents for the generation of new realities that represent gender binary breakdowns, precisely because of their ability to rewrite anatomies of possible bodies as well as social norms around these bodies.

The future-oriented narratives of gender hacking are, centrally, a challenge against dominant medical narratives around who can or should control the use and development of biomedical technologies like synthetic hormones. Through gender hacking, hormone technologies are harnessed for political purposes other than those for which they were initially designed in biomedical contexts. Notably, gender hacking is connected with radical forms of trans health activism that call for free, unconstrained assess to hormones. This includes enabling self-medication for gender affirming purposes, where self-medication refers to self-administering hormones—often accessed via means such as online (sometimes black market) pharmacies and hormone exchange community groups—not prescribed by medical professionals. For example, the Edinburgh Chapter of Action for Trans Health (2017), a grassroots organisation advocating for the democratisation of trans and non-binary healthcare, have manufactured a “vision for trans futures”, narrated in a manifesto that envisions.

free, universal access to safe hormones & blockers at any age, the opportunity to decide our own doses, and universally accessible information of the safety & efficacy of different regimes. … We demand the freedom to alter our bodies without justification.

These expressions of trans health activism are contextualised by the wider structural and regulatory conditions that constrain access to gender-affirming hormones for trans and non-binary people via mainstream healthcare systems. As Pearce (2018) among others has documented, accessing gender-affirming hormones via ‘authorised’ means today generally poses significant, sometimes insurmountable challenges, because medical professionals act as gatekeepers of hormones, controlling access. Access is generally mediated through multiple appointments and extensive assessment processes and is conditioned by one’s ability to meet particular clinical treatment criteria, with healthcare professionals having the final say on who is (not) given access and when. Concurrently, medically oriented studies that examine the phenomenon of self-medication for gender affirming purposes frame it as potentially dangerous high-risk behaviour, representing individuals engaged in it as vulnerable and insufficiently informed to understand the medical implications of self-medicating (e.g. Mepham et al. 2014; Rotondi et al. 2013; Sanchez et al. 2007). These narratives also implicitly position professional medical practitioners as the correctly informed ‘experts’ and thus as the appropriate gatekeepers of hormones, rendering promissory ‘vision for trans futures’ advocated by groups like Action for Trans Health as potentially harmful rather than emancipatory.

In this context, gender hacking and radical trans health politics manufacture interlinked and overlapping narratives of resistance against structural constraints and medical gatekeeping, envisioning futures other than those where medical professionals regulate the ‘appropriate’ gendered realities and ways bodies can be altered. While mainstream medical narratives configure hormones as substances that should be controlled and managed by medical professionals due to their dangerous nature, in the narratives of gender hackers and radical trans health activists, hormones act as agents of resistance. They become key enablers of gender emancipatory futures, which presume that hormones need to be liberated from the constraints and gatekeeping that they are currently subject to within healthcare systems so that they can carry out gender emancipatory work at the intersection of bodies and the social contexts in which they are made sense of. Hormones thus act as central agents in politically loaded struggles over what the future of gender should look like, and how emancipatory futures for gender diverse people could and should be realised. In a space where utopian dreams intermingle with the need for simple survival within institutional and structural constraints, hormones play a central role in promissory future-oriented visions for gendered bodies and social relations beyond the binary.

## Hormones as emploting ‘the natural’ and ‘the medicalised’: birth hormones and childbearing bodies

Ford’s 2013–2016 fieldwork on childbearing in California illustrates how, in narratives that position hormones—specifically, oxytocin—as key protagonists in the processes of childbirth, hormones come to embody and re-articulate an enduring tension between ‘the natural’ and ‘the medicalised’ within wider politics around childbirth. In this context, birth hormones and the cascades of effects they can incite inside and outside the body shape not only how bodies (are understood to) work but how they are necessarily in relation to places and people that make up their social, physical, and emotional contexts.

Childbearing and birth are replete with dramatic narratives—from personal narratives about the experience, be it traumatic or transformational (Pollock 1999), to competing cultural stories about the role of institutional medicine. The dominant narrative about institutional medicine, which it itself reproduces, is a story of technological progress and triumph over death and pain, including the ‘dangerous’ process of childbirth. This is opposed by the underdog story of a patriarchal medical takeover that stole birth from the midwives and women to whom it formerly belonged (e.g. Ehrenreich and English 1973), and that manufactures the dangers it claims to prevent (e.g. Davis-Floyd and Cheyney 2009). However, in the narratives of Ford’s research participants, these old power struggles were rehashed in new language: the language of hormones, where birth hormones were repeatedly emplotted as a central actor in childbearing. Stories centring oxytocin and the related hormonal cascades of ‘safety’ or ‘stress’ surfaced everywhere from doula training, to birthing people explaining their homebirth rationale, to hospital nurses turning the lights down during labour, to the small-but-growing medical critique of labour induction. Indeed, oxytocin has developed a bit of a cult following in birth worlds: its molecular structure makes appearances everywhere from “Birth Chemistry” childbirth education programmes, to necklaces sold on the bespoke website Etsy, to pop-science books like *Oxytocin: The Biological Guide to Motherhood* (Uvnäs-Moberg 2016). Countless birth blogs extoll its virtues, and it features in memes and cartoons.

The basic story went like this: oxytocin is a key actor responsible for causing uterine contractions, progressing labour, providing pain relief, initiating breastfeeding, and stimulating bonding between birthing person and infant. This role is largely uncontested in medical and non-medical discourses. Yet, the extent to which oxytocin can carry out this role is dependent on the wider environment in which childbirth is occurring because the environment shapes how oxytocin works in the body. Those who advocate for less medicalisation of birth position medicalised environments as inhibitors of oxytocin’s ‘natural’ role during childbirth and emphasise that feeling safe enables the oxytocin system to work, while feeling threatened produces adrenaline, inhibiting the oxytocin response. Oxytocin is able to work when a person is in a calm, protected, familiar environment where they do not have to think, and can relax into a state of intensity, focus, and instinct. Meanwhile, cortisol and adrenaline, the ‘fight, flight, or freeze’ hormones that are mutually reinforcing with stress and fear, are produced in environments with strangers, bright lights, lots of questions, and feelings of risk—like most hospitals.

In these stories, oxytocin and cortisol/adrenaline come to form the basis of two ‘cascades’, or cycles of effects that are initiated by the hormones’ actions, one positive and one negative. The more relaxed a person is, the easier it is for oxytocin to work, and the faster their birth will be. The more fear and stress present in the birth room, the more labour processes will be stalled, and the more medical interventions will be needed, further increasing fear and stress. Synthetic oxytocin—the drug Pitocin—is commonly used in hospitals to start labour, increase contractions’ intensity, and deliver the placenta. But those who champion reduced medical interference emplot Pitocin as an agent of medicalised birth environments and emphasise that it acts to interrupt the ‘natural’ production and function of oxytocin, effectively causing a third kind of cascade: the cascade of interventions. In this cascade, inducing labour with Pitocin inhibits the body’s production of oxytocin, which means more Pitocin will be required for labour to progress, which means the contractions will be very strong and painful because Pitocin does not stimulate pain relief as oxytocin does. This will lead to an epidural for pain relief, but the foetus will still feel the hyper-strong contractions and may exhibit distress because of them, which is cause for further Pitocin to speed delivery, often leading to caesarean surgical delivery. Finally, in this cascade, the lack of oxytocin makes breastfeeding and bonding between the new parent and infant challenging, potentially leading to postpartum depression and formula feeding, which are associated with their own chains of negative effects.

According to these oxytocin narratives, medical spaces, personnel, and procedures are all implicated in triggering undesirable hormonal cascades. In recent decades, American hospitals have attempted to become more ‘home-like’ to appease the many critiques levelled against hospital birth. Changes include welcoming family members and doulas (people trained to give birth support), inviting patients to bring their own pillows, lighting, and music, and otherwise crafting an environment that puts patients at ease. Crafting the atmosphere in this way goes alongside more fundamental shifts that de-prioritise technology and medical convenience, such as not requiring an IV or continuous foetal monitor, not pressuring people to have an epidural for pain relief (without one, they can move around and deliver without lying on their back) and hesitating to induce or augment labour with Pitocin.

These shifts connect oxytocin cascades in the body and the influence carried by the immediate environment of childbearing to wider politicised tensions between ‘natural’ versus ‘medicalised’ childbirth—or rather, these new narratives re-articulate this old division, swapping hormones and the biological realities they are taken to represent for more forthrightly political motivations like respect and autonomy. Over the past half-dozen decades, American birth activism has organised prominently around narratives of feminism and human rights, notably in the natural birth movement in the 1970s and the professionalisation of midwifery that followed in the 1990s, both largely white, middle-class initiatives (see Craven 2007; Kline 2016; MacDonald 2018), while in other communities, medical intervention and less-medicalised approaches to birth have distinct, albeit intersecting, histories that emphasise racism and abuse, access and inclusion, and broader projects of community justice (e.g. Fraser 1995; Roberts 1997; Ross and Solinger 2017; Davis 2019; Scott and Davis 2021). Very recently, narratives about evidence have started to feature in activism in movements for ‘evidence-based birth’ that reclaim biomedicine’s own language and ideals within different political configurations (Ford 2019; Akrich et al 2014). Stories centring hormones’ role in childbirth fall into this pattern, linking qualitative aspirations with institutionally powerful narratives about science, increasing these activist stories’ traction while limiting their scope.

People concerned with dominant models of birthing recruit hormones as central protagonists to explain why physiology is on their side. In doing so, they borrow from medical narratives about empirical evidence and the primacy of physio-chemical reality, but advocate for practices that push against medical protocols and power dynamics centred around doctors. There is power in making use of dominant scientific narratives in this way, as it links political goals with socially accepted and empowered forms of knowledge. However, narratives formerly used for the purpose, including those about social justice, feminism, human rights, and empathy, risk falling by the wayside, leaving power dynamics as such unnamed and unchallenged. The transformative potential of politicising birth-room practices is curtailed, even while positioning hormones as key actors in childbearing narratives facilitates immediate and practical changes.

Stories about hormones as arousing embodied cascades during childbearing also link up with narratives about self-optimisation and self-knowledge in privileged contexts where cultivating oneself is both an aspiration and source of intense pressure, a means of empowerment and source of alienation (Ford 2020). In such contexts, hormone function is simultaneously emplotted as an involuntary biological response and something one can influence, for example through crafting a birth environment or adopting a certain attitude. One must gather knowledge about biology, socio-medical systems, and one’s feelings and identity in order to produce oneself in this way in relation to hormones’ actions, including by crafting an environment where oxytocin can cascade positively. Such narratives about self-realisation through the intense experience of birth do grant a large degree of agency to the birthing person as well as to oxytocin and do so while minimising narratives about appropriately gendered behaviour. Yet, they focus on the empowered individual at the expense of the broader socio-political dynamics of privilege and alienation.

Narratives where birth hormones play central roles in childbearing thus emplot these hormones as key actors in the politics of birth. Oxytocin becomes representative of, and a protagonist in, ‘natural’ childbirth while medicalised environments—including synthetic hormonal agents like Pitocin—come to inhibit oxytocin’s actions in ways that represent the dangers of medicalisation. Hormones can initiate both positive and negative cascades depending on the environment in which their actions are manifesting, lending biological credibility to narratives about ‘good’ environments. Oxytocin and its supporting cast thus create cascades not only in bodies but in their spatial surroundings and the broader political landscapes. Narratives that position hormones as central actors have political stakes not just because of what they make possible but because of what they obscure. The narratives, as well as the hormones, cascade into one another, evolving and replacing each other, and responding to broader cultural shifts.

## Hormones articulating sensory—limbic experience: complicating dominant narratives of autism

During Malcolm’s long-term participant observation in the UK and USA with autistic children, adults, and their families, and practitioners of an autism-specific form of horse therapy, she encountered stories that exemplify how hormones come to be framed as environmentally situated and relational agents. As shown below, these stories situated autistic experience in affective and architectural environments, embodied sensory difference, “fight or flight” responses and hormonal flows in the blood (Malcolm 2019, 2021). This model reframed autism as an embodied, emplaced (Howes 2005) condition enacted in social relations rather than a disorder of the individual body. It decentred the neurological as the site of the condition. Speaking to the key thread of argumentation weaving our patchwork of stories together, hormones, via their role in the “fight or flight” response were key actors in this reframing of autism. Through detailing the hacking of hormones, [Author’s name]’s practitioner interlocutors engaged with authoritative biological models of the body, yet simultaneously challenged simple biologisation of the condition.

Stories representing the lives of one very specific kind of (‘high functioning’, savant) autistic person have abounded in film and television over the last few decades (Murray 2008). Yet as the saying goes, when you know one autistic person, you know one autistic person. More recently, the burgeoning genre of autistic autobiography has given increased public access to the lived experience of the condition. This communicates an emerging narrative of being autistic that foregrounds embodied sensory difference. Autistic self-advocacy and neurodiversity movements have grown in strength, advocating for the reconfiguration of autism as a form of difference, a way of being in the world. Indeed, after years of lobbying, the Autism Self-Advocacy Network (ASAN) successfully advocated for sensory idiosyncrasies to be included in the Diagnostic and Statistical Manual of Mental Disorders (DSM-5) that defines the condition for the first time. This was a positive move in acknowledging autistic embodiments, yet sensory experience arguably entered the DSM-5 (APA 2013) criteria framed as a form of pathology. This highlights how advocates must negotiate within the parameters of narratives they may seek to trouble, something reflected in how hormones were mobilised in the context of horse therapy.

Some stories about autism are taken up and promoted widely, and others silenced. As a category, ‘autism’ continues to be bound up in the configuration of ‘normal’ and ‘pathological’ forms of sociality and communication within authoritative biomedical, and lay, knowledges (Hollin 2014) affecting which stories circulate more easily than others. The well documented—and ongoing—tensions between neurodiversity and deficit models of the condition (Broderick and Ne’eman 2008) constrain the flows of particular knowledges, ultimately affecting to what end such stories are mobilised—and arguably who or what becomes responsibilised. For the practitioners that [Author’s name] got to know, hormonal cascades within the body became powerfully salient. This was via their ability to articulate the effects of particular sensory architectures on “fight or flight systems” that some of [Author’s name]’s autistic interlocutors used as a way to articulate their experience of being autistic.

Practitioners narrated the perceived therapeutic efficacy of equine therapy in getting to know the “good” and “bad sensory triggers” of each client, before carefully choreographing calming horseback embodiments and environments around these particular individual sensitivities. Another scale was added to this therapeutic ecology by imbuing it with the aura of hormonal flows that biologised and molecularised the condition. Heightened sensory sensitivities could lead to activation of the “fight, flight or freeze” response. After which, as practitioner and trainer, Amy puts it.

the amygdala is activated. Danger, danger, danger. Cortisol, cortisol, cortisol. At a certain point, if the amygdala is being continually activated… this person will start to experience too high levels of cortisol.

Here, the practitioner chimes with what scholar and autistic advocate, Damian Milton, has called the “negative spiral of stress” (2013). Oxytocin was perceived to be able to counteract these flows.

As Amy puts it, and in a particularly moralising tone, “cortisol is our enemy, and oxytocin is our friend”. For Amy, riding horses was so beneficial for autistic children “because of the oxytocin being produced”. Hormones were being hacked via horses’ movement and enabling more sensorially sensitive environs. Rebalancing cortisol and oxytocin flows was not assumed to offer any kind of ‘fix’ or state of constancy, but an ongoing, weekly space for calm and embodied communication through the provision of more sensitively designed affective and architectural spaces.

As we argue here and in line with work elsewhere (Malcolm et al., forthcoming), hormones are key protagonists in how we understand not only the body in health and ill-health, but experiences of self and relations to others. Appeals to the “fight or flight” response (a central component in the excitation of hormonal flows) are increasingly common in lay narratives about health; particularly as ways to articulate stress and burn out. Emplotted in the context of an autism-specific method of horse therapy (AM) were stories about a particularly active fight or flight response in autistic people resulting from sensory sensitivities. This response was understood to be triggered by unpredictable environments and sensorially toxic spaces such as schools and supermarkets populated with continually flashing strip lighting, loud unpredictable noises, and chemical odours. In the context of horse therapy, this emerging emplaced, hormonal story is situated within a ‘therapeutic ecology’ co-constructed in varied ways by practitioners and autistic participants of horse therapy (Malcolm 2021).

This gives an entry into the complex model of autism being enacted through the practices of equine therapy. That is, the mobilisation of biomedical knowledges in articulating kinds of difference and the resonances across scales of the situated body-person-world (Latimer 2011) bound up in interlocutors’ perceptions of therapeutic efficacy. Efficacy was crafted by practitioners around an understanding of autism as a deeply embodied condition, and one enacted through the inhabited environment (Malcolm 2019). Hormones were emplotted as the fluid connection between various scales of the body required to articulate such a complex model of the body and self in relations with other selves and environments. Their role was to provide a nexus for the body-person-world.

For the autistic and non-autistic people in this context, autistic experience was not situated in individual brains, it involved multiple interrelated processes of the mind/body/brain and environment. While this sensory-limbic model was used by Malcolm’s autistic interlocutors, they did not tell hormonal stories about autism. Yet, practitioners of AM appealed to the authoritative language of hormones, stating that hormones played a key role in wellbeing for autistic children and adults and tinkering with hormonal flows could enhance wellbeing. The idea of hormonal cascades offered a powerful heuristic to encapsulate the complexly shifting, looping scales and partial connections of body-person-worlds (Malcolm 2019; Latimer 2011) mobilised in this story of autism—and relatedly therapeutic efficacy—being told in relation to and through the actions of hormones.

Autistic ways of being were narrated as a product of an interplay of endocrine, peripheral, and central nervous systems of the body emplaced with the built and social environment. The bounded, individual ‘body proper’ therefore fails to offer a comprehensive account of local understandings of the phenomenon in focus. As Haraway asks “why should the body end at the skin?” (1991, p. 22). To make the body a topic for anthropological and sociological inquiry today is to ask how it is lived; how is it constructed, imagined, and subjectively known (Lock and Farquhar 2007). The body was subjectively known as irreducibly situated in a heightened worldly sensitivity that Manning (2013) calls autistic perception. This was articulated via processes of the limbic system and the “fight, flight, freeze” response. Yet, through these authoritative biomedical knowledges, a clear message was conveyed that environments played as significant a role in the creation of anxiety, and ultimately chronic stress experienced by autistic people, speaking to what Shakespeare and Watson (2001) have termed an embodied ontology of disability.

As we have noted, hormones are defined by their activity and named in reflection of their role ‘to excite’ and ‘set in motion’. They flow and enact relations and intra-actions (Barad 2007), not only of bodily processes but also situated embodiments. This very property of movement, alongside their increasing salience in popular understandings of the body, allowed hormones to flow in and provide an alternative pool for reflection on autistic experience, moving across scales of inside and outside, self and other, and articulating the situated character of the ongoing anxieties and stress all too often lived by autistic people. Stories with hormones as key protagonists—building on highly salient contemporary narratives of “fight or flight”—were used to communicate the sensory-perceptual situatedness of one’s lived experience, yet these stories simultaneously identified these phenomena in biologised and individualised bodily flows, reproducing normalising molecular narratives around autistic ‘malfunction’. This highlights complexities and tensions emerging through the telling of hormone stories, the normative constructions of the pathological reproduced, yet also the novel bodily analogies articulated as a form of resistance to authoritative knowledges enacted through and with hormones.

## Conclusion

While hormones were historically conceptualised as ‘master molecules’ representing the prospect of controlling central life processes, from reproduction to respiration to sleep, the technoscientific and socio-cultural context of today has fundamentally shifted how hormones are understood and what roles they perform in the world. In the last few decades, the ‘master molecule’ vision of hormones has arguably been backgrounded with new and emerging biomedical developments such as neurotransmitters, gene-based biotechnology and omics. Yet, drawing from empirical data from four distinct contexts, we argue that hormones and hormone stories are in ascendency, as hormones increasingly feature in stories and practices that have profound influence on people’s lived experiences and inhabited worlds (see Horgan and Dickson 2020). These hormone stories challenge dominant biomedical models of bodies and expectations around how people ‘fit’ within dominant models of bodily experience, and they articulate how biological bodies, forms, and processes become situated in their various environs. By building on new materialist frameworks that configure hormones as active and agentic entities that ‘provoke’ biosocial phenomena, including narratives that build meaning into the world, we have explored the new kinds of protagonists that hormones are today in stories told about bodies, selves, and the environments they inhabit. In so doing, we have shown that hormones actively participate in the narrative emplotment of reality through which meaning is generated, including by arousing or putting into action successions of events that form (and are interpreted as) a plot.

By bringing together hormone stories from four different research projects, we have woven together a thread that highlights not only the multifaceted narrative provocations that hormones incite today, but also how these narrative provocations do diverse kinds of social and biological, personal, and political work. Across these four research projects, hormones sit at the intersection of dominant biomedical narratives and alternative narratives that attribute different roles to them than those posited by medical discourses. In these contexts, hormones are mobilised to hack bodies, and in so doing hack notions of what particular bodies are or can be. Hormones are thus the subjects of multiple contested stories in which they act as both agents of and sites of resistance against the medicalisation of bodies and behaviours. In these new kinds of stories, using or not using hormonal technologies and hacking into or tinkering with hormones is positioned as enabling an intervention into biological or embodied processes as well as social, cultural, and political ones. Hormones are thereby made protagonists in and across the overlapping realms of the personal and the political. They act as catalysts for cascades of effects that flow in and through the body in environmentally situated, relational ways. Contemporary hormonal narratives make hormones into links connecting different realms of life—body, self, societybiology—as they multiply and provoke.

In the Swedish women’s accounts about hormonal contraceptives, hormones play central roles in multiple narratives of ‘control’ that were invoked and mobilised in relation to hormonal contraceptives as a gendered technology that acts on the body to arouse physical effects and to shape one’s sense of self in affective ways. While the women drew from dominant biomedical narratives that position hormonal contraceptives as an empowering technology that enables control over women’s presumed fickle reproductive bodies and risky fertility, they also challenged and undermined these dominant narratives. In doing so, they produce alternative conceptualisations of what ‘control’ in the context of contraception is or should entail, shaped by hormones’ ability to act on the body in undesired as well as in desired ways. These contraceptive stories and experiences highlight the complex material-discursive processes through which hormonal enactments and contraceptive meanings are made, and remade.

Conversely, in the promissory future-oriented narratives emplotted by ‘gender hackers’ and radical trans health activists, hormones invoke emancipatory gender politics and better future worlds where hormones act as sites of empowerment that should be liberated from the sphere of control that medical institutions and gatekeepers currently exercise over them. Like the Swedish women’s narratives of hormonal contraceptives, gender hacking narratives frame control over hormone technologies away from authoritative medical narratives about gendered bodies, but here hormones act explicitly as subversive protagonists that can be harnessed to resist medicalised control. They are attributed with the power to ‘hack’ into the medicalised binary gender system to re-make bodies and social relations beyond the gender binary, where their subversive potential is attached both to their ability to alter bodies and, relatedly, to their potential to alter the future of gender as a social, cultural and political system.

Relatedly, in the hormonal plots of Californian birthing cultures, biomedical stories about how birth hormones work in the body merge with politicised stories about what kinds of environments are best suited to birth and how the experience of giving birth should unfold. In these stories, birth hormones and the cascading effects they can arouse across the body, birthing self, and wider environment come to represent either natural or medicalised childbirth depending on how and where the birth happens. Hormones’ actions are framed as conditioned by the immediate environment in which childbirth occurs, which is, in turn, linked with the wider politics of childbirth. In this way, hormones cascade across the personal and the political, forming a web of links between the body and the wider context in which it is embedded. In the context of autism-specific horse therapy, hormones come to sit at the nexus of body-person-worlds, articulating sensory-limbic experiences linking the body and the self with their wider environment through acting as mediators of a social ontology of autistic experience, human–horse and human–environment relations. While autistic experience itself is emplotted as involving multiple interrelated processes of the mind, body, and brain, hormones took centre stage at the crux of these processes. Biomedical narratives of both autism and hormones were rendered authoritative, yet they were complicated by and mediated through individual experiential, embodied, and environmentally situated stories that re-directed the flow of hormones to these wider ecologies.

Collectively, the above patchwork illustrates that hormones continue to be vital protagonists in the stories that are told to make sense of the world today, even while the ‘master controller’ narratives have been complicated by more diffused and nuanced stories of hormones. In the contemporary world where hormones flow with phenomena like genes and omics in the narrative emplotment of life, they emerge in sites of resistance where bodies are hacked, and society and politics are tinkered with. Hormones mediate dynamics of dominant and alternative narratives about bodily experience. They flow and incite cascades across the biomedical, socio-cultural, environmental, personal, political, and regulatory spheres of life, mediating their nexus.

## Data Availability

The participants of the studies on which this paper is based did not give written consent for their data to be shared publicly, so due to the sensitive nature of the research, supporting data is not available.

## References

[CR1] Ah-King, Malin, and Eva Hayward. 2013. Toxic sexes: Perverting pollution and queering hormone disruption. *O-Zone: A Journal of Object-Oriented Studies* 1: 1–12.

[CR2] Akrich, Madeleine, Máire. Leane, Celia Roberts, and João Arriscado. Nunes. 2014. Practising childbirth activism: A politics of evidence. *BioSocieties* 9 (2): 129–152.

[CR3] APA. 2013. *Diagnostic and statistical manual of of mental disorders: DSM-5*, 5th ed. Arlington: American Psychiatric Publishing.

[CR4] Barad, Karen Michelle. 2007. *Meeting the universe halfway: Quantum physics and the entanglement of matter and meaning*. Durham, N.C.: Duke University Press.

[CR5] Bertotti, Andrea, Emily Mann, and Skye A. Miner. 2021. Efficacy as safety: Dominant cultural assumptions and the assessment of contraceptive risk. *Social Science & Medicine* 270: 113547. 33455813 10.1016/j.socscimed.2020.113547

[CR6] Broderick, Alicia, and Ari Ne´eman. 2008. Autism as metaphor: Narrative and counter-narrative. *International Journal of Inclusive Education* 12 (5–6): 459–476.

[CR7] Brown, Nik, Brian Rappert, and Andrew Webster. 2000. *Contested futures: A sociology of prospective techno-science*. Aldershot: Ashgate.

[CR8] Craven, Christa. 2007. A “consumer’s right” to choose a midwife: Shifting meanings for reproductive rights under neoliberalism. *American Anthropologist* 109 (4): 701–712.

[CR9] Davies, S. 2017. *Hackerspaces: Making the maker movement*. Cambridge: Polity Press.

[CR10] Davis, Dána-Ain. 2019. *Reproductive injustice: Racism, pregnancy, and premature birth*. New York: NYU Press.

[CR11] Davis-Floyd, Robbie, and Melissa Cheyney. 2009. Birth and the big bad wolf: An evolutionary perspective. In *Childbirth across cultures*, ed. Helaine Selin and Pamela K. Stone, 1–22. Dordrecht: Springer.

[CR12] Delfanti, A. 2013. *Biohackers: The politics of open science*. London: Pluto Press.

[CR13] Delgado, A. 2013. DIYbio: Making things and making futures. *Futures* 48: 65–73.

[CR14] Dickinson, Adam. 2018. *Anatomic*. Toronto: Coach House Books.

[CR15] Dickinson, Adam. 2019. Outside inside. *Environmental Humanities* 11 (1): 174–179.

[CR16] Edinburgh Chapter of Action for Trans Health. 2017. Trans health manifesto. https://edinburghath.tumblr.com/post/163521055802/trans-health-manifesto. Accessed 8 Sep 2020

[CR17] Ehrenreich, Barbara, and Deirdre English. 1973. *Witches, midwives, and nurses: A history of women healers*. New York: The Feminist Press.

[CR18] Erikainen, S. 2019. *Gender verification and the making of the female body in sport: A history of the present*. New York: Routledge.

[CR19] Erikainen, S., A. Ford, R. Malcolm, and L. Raeder. 2020. Telling hormonal stories. In *So hormonal*, ed. E. Desmont and L. Nickodemus. Edinburgh: Mostrous Regiment Publishing.

[CR20] Fausto-Sterling, Anne. 2000. *Sexing the body: Gender politics and the construction of sexuality*, 1st ed. New York: Basic Books.

[CR21] Ford, Andrea. 2019. Advocating for evidence in birth: Proving cause, effecting outcomes, and making the case for ‘curers.’ *Medicine Anthropology Theory* 6 (2): 25–48. 37155520 10.17157/mat.6.2.674 PMC7614474

[CR22] Ford, Andrea. 2020. Birthing from within: Nature, technology, and self-making in silicon valley childbearing. *Cultural Anthropology* 35 (4): 602–630. 37113568 10.14506/ca35.4.05 PMC7614481

[CR23] Fraser, Gertrude. 1995. *Modern bodies, modern minds: Midwifery and reproductive change in an African American Community. in Conceiving the new world order: The global politics of reproduction*. Berkeley: University of California.

[CR24] Granzow, Kara. 2007. De-constructing ’choice’: The social imperative and women’s use of the birth control pill. *Culture Health & Sexuality* 9 (1): 43–54. 10.1080/13691050600963948 17364713

[CR25] Hackteria. 2019. Open source estrogen. http://www.hackteria.org/wiki/index.php?title=Open_Source_Estrogen&oldid=34158. Accessed 9 Sep 2020

[CR26] Haraway, Donna. 1991. *Simians, cyborgs, and women: The reinvention of nature*. New York: Routledge.

[CR27] Haraway, Donna. 1997. *Modest-Witness@Second_Millenium. FemaleMan_Meets_OncoMouseTM*. New York: Routledge.

[CR28] Hird, Myra. 2019. Material feminist engagements. *Feminist Studies* 35 (2): 329–346.

[CR29] Hollin, Greg. (2014). Autism, sociality and human nature. *Somatosphere*. June 18.

[CR30] Horgan, Emily, and Zachary Dickson. 2020. *So hormonal: A collection of essays on hormones*. Edinburgh: Monstrous Regiment Publishing.

[CR31] Howes, David, ed. 2005. *Empire of the senses: The sensual culture reader*. Oxford: Berg.

[CR32] Irni, Sari. 2013. Sex, power and ontology: Exploring the performativity of hormones. *Nora* 21 (1): 41–56.

[CR33] Jordan-Young, Rebecca, and Katrina Karkazis. 2019. *Testosterone: An unauthorized biography*. Cambridge: Harvard University Press.

[CR34] Kirby, Vicky. 1997. *Telling Flesh: The Substance of the Corporeal*. New York: Routledge.

[CR35] Kline, Wendy. 2016. Chapter 6: The little manual that started a revolution: How hippie midwifery became mainstream. In *Groovy science: Knowledge, innovation, and American counterculture*, ed. David Kaiser and Patrick W. McCray, 172–204. University of Chicago Press.

[CR36] Laboria Cubonics. 2015. Xenofeminism: A politics of alienation. https://laboriacuboniks.net/manifesto/xenofeminism-a-politics-for-alienation/. Accessed 9 Sep 2020

[CR37] Langston, Nancy. 2010. *Toxic bodies: Hormone disruptors and the legacy of DES*. New Haven: Yale University Press.

[CR38] Latimer, Joanna. 2011. Home, care and frail older people: Relational extension & the art of dwelling. In *Perspectives on care at home for older people*, ed. Christine Ceci, Mary Ellen Purkis, and Kristin Björnsdottír, 35–61. New York: Routledge.

[CR39] Lock, Margaret, and Judith Farquhar. 2007. *Beyond the body proper: Reading the anthropology of material life*. Durham: Duke University Press.

[CR40] MacDonald, Margaret E. 2018. The making of informed choice in midwifery: A feminist experiment in care. *Culture, Medicine, and Psychiatry* 42 (2): 278–294. 29143236 10.1007/s11013-017-9560-9

[CR41] MacKendrick, Norah, and Kate Cairns. 2019. The polluted child and maternal responsibility in the us environmental health movement. *Signs: Journal of Women in Culture and Society* 44 (2): 307–332.

[CR42] Malcolm, Roslyn. 2019. *Rhythms that matter: The kinetic melodies and matterings of autism and equine therapy practices in the UK and USA.* Doctoral Thesis. University of Edinburgh.

[CR43] Malcolm, Roslyn. 2021. “There’s no constant”: Oxytocin, cortisol and balanced proportionality in hormonal models of autism. *Medical Anthropology* 40 (4): 375–388. 36018784 10.1080/01459740.2021.1894558

[CR44] Malcolm, R., S. Erikainen, A. Ford, L. Raeder, and C. Roberts. Hormonal theory: Introduction. In *Hormonal theory: A critical glossary*, ed. A. Ford, R. Malcolm, S. Erikainen, L. Raeder, and C. Roberts. Bloomsbury, forthcoming.

[CR45] Mamo, Laura, and Jennifer R. Fosket. 2009. Scripting the body: Pharmaceuticals and the (re)making of menstruation. *Signs Journal of Women in Culture and Society* 34 (4): 925–949.

[CR46] Manning, Erin. 2013. *Always more than one: Individuation’s dance*. Durham: Duke University Press.

[CR47] Martin, Emily. 1991. The egg and the sperm: How science has constructed a romance based on stereotypical male-female roles. *Signs* 16 (3): 485–501.

[CR48] Martin, Emily. 2001. *Flexible bodies: Tracking immunity in American culture from the days of polio to the age of AIDS*. Boston: Beacon Press.

[CR49] Mattingly, C. 1994. The concept of therapeutic emplotment. *Social Science & Medicine* 38 (6): 811–822. 8184332 10.1016/0277-9536(94)90153-8

[CR50] Mepham, N., W. Bouman, J. Arcelus, M. Hayter, and K. Wylie. 2014. People with gender dysphoria who self-prescribe cross-sex hormones: Prevalence. *Sources, and Side Effects Knowledge, Journal of Sexual Medicine* 11: 2995–3001. 25213018 10.1111/jsm.12691

[CR51] Milton, D. E. M. 2013. Reversing the negative spiral of stress—A personal and philosophical reflection. In *Stress and autism: Combating stress, lightening the load, research autism conference*, London, UK, May 14th (**Unpublished**).

[CR52] Murray, Stuart. 2008. *Representing autism: Culture, narrative, fascination*. Liverpool: Liverpool University Press.

[CR53] Nordlund, C. 2007. Endocrinology and expectations in 1930s America: Luis Berman’s ideas on new creations in human beings. *BJHS* 40 (1): 83–104. 17575959 10.1017/s0007087406009113

[CR54] Oudshoorn, Nelly. 1994. *Beyond the natural body: An archaeology of sex hormones*. London: Routledge.

[CR55] Pearce, Ruth. 2018. *Understanding trans health: Discourse, power, and possibility*. Bristol: Policy Press.

[CR56] Plummer, Kenneth. 1995. *Telling sexual stories: Power, change and social worlds*. London: Routledge.

[CR57] Pollock, Anne. 2016. Queering endocrine disruption. In *Object-oriented feminism*, 1st ed., ed. Katherine Behar, 183–199. Minneapolis: University of Minnesota Press.

[CR58] Pollock, Della. 1999. *Telling bodies performing birth: Everyday narratives of childbirth*. New York, NY: Columbia University Press.

[CR59] Preciado, Paul B. 2018. *Testo junkie: Sex, drugs and biopolitics in the pharmacopornographic era*. Feminist Press: City University of New York.

[CR60] Rasmussen, N. 2002. Steroids in arms: Science, government, industry, and the hormones of the adrenal cortex in the United States 1930–1950. *Medical History* 46: 299–324. 12194423 PMC1044526

[CR61] Reed, Lori Stephens, and Paula Saukko, eds. 2010. *Governing the female body: Gender, health, and networks of power*. Albany: State University of New York Press.

[CR62] Roberts, Celia. 2007. *Messengers of sex: Hormones, biomedicine and feminism*. Cambridge: Cambridge University Press.

[CR63] Roberts, C., and C. McWade. 2021. Messengers of stress: Towards a cortisol sociology. *Sociology of Health and Illness* 43: 895–909. 34056738 10.1111/1467-9566.13261

[CR64] Roberts, Dorothy. 1997. *Killing the black body: Race, reproduction, and the meaning of liberty*. New York: Pantheon Press.

[CR65] Ross, Loretta, and Rickie Solinger. 2017. *Reproductive justice: An introduction*. Oakland: University of California Press.

[CR66] Rotondi, N. 2013. Nonprescribed hormone use and self-performed surgeries: “Do-it-yourself” transitions in transgender communities in Ontario. *Canada, American Journal of Public Health* 103 (10): 1830–1836. 23948009 10.2105/AJPH.2013.301348 PMC3780733

[CR67] Sanchez, N., J. Sanchez, and A. Dandoff. 2007. Health care utilization, barriers to care, and hormone usage among male-to-female transgender persons in New York City. *American Journal of Public Health* 99 (4): 713–719. 10.2105/AJPH.2007.132035 PMC2661470 19150911

[CR68] Scott, Karen A., and Dána-Ain. Davis. 2021. Obstetric racism: Naming and identifying a way out of black women’s adverse medical experiences. *American Anthropologist* 00: 1–4.

[CR69] Shakespeare, Tom, and Nicholas Watson. 2001. The social model of disability: An outdated ideology? *Research in Social Science and Disability* 2: 9–28.

[CR70] Smith, Brett, and Andrew C. Sparkes. 2011. Exploring multiple responses to a chaos narrative. *Health (london, England)* 15 (1): 38–53. 10.1177/1363459309360782 21212113

[CR71] Uvnäs-Moberg, Kerstin. 2016. *Oxytocin: The biological guide to motherhood*. San Francisco: Praeclarus Press.

